# Protein-Bound Azo Dyes in the Serum of Rats Fed 3'-Methyl-4-Dimethylaminoazobenzene

**DOI:** 10.1038/bjc.1961.22

**Published:** 1961-03

**Authors:** J. Dijkstra, F. J. Joubert


### PROTEIN-BOUND AZO DYES IN THE SERUM OF RATS FED 3′-METHYL-4-DIMETHYLAMINOAZOBENZENE

J. DIJKSTRA AND F. J. JOUBERT

*From the National Chemical Research Laboratory, South African Council for Scientific and Industrial Research, Pretoria*

Received for publication January 20, 1961

SEVERAL aminoazo dyes are known to induce liver tumours when fed to rats (Miller and Miller, 1953, 1955). The dyes which appear in the liver after feeding have been classified as " free ", " polar bound " and " non-polar bound " by Miller and Miller (1947). The free dye is that fraction which can be extracted by ethyl alcohol from the dried tissue protein. The bound dye, in contrast, cannot be removed from the intact proteins by even the most rigorous extraction, but is only liberated after alkaline hydrolysis. The non-polar fraction of the bound dye can be extracted from the hydrolysis mixture by petroleum ether, whereas the polar fraction, which constitutes the major part, can be extracted only by an ethyl alcohol-ethyl ether mixture. The non-polar dyes have been identified as N-demethylation products, while the polar bound dyes are believed to owe their polar properties to their attachment to some amino acid or peptide fragment released from the liver protein by the alkaline hydrolysis (Miller and Miller, 1952, 1953, 1955 ; Terayama, Ishidate and Hanaki, 1959).

In rats fed aminoazo dyes, either continuously at a low level, or in a single large dose, the only organ in which bound dye is found is the liver (Miller and Miller, 1947 ; Gelboin, Miller and Miller, 1958). Studies on the accumulation of protein-bound dye in the liver under various conditions indicate that the formation of this bound dye has a role in the carcinogenic process, possibly through deletion of the protein involved in the binding (Miller and Miller, 1947, 1953, 1955 ; Miller *et al.*, 1949). However, the observation that high levels of bound dyes are found in the livers of rats fed the weakly carcinogenic 2-methyl-4-dimethylaminoazobenzene and 3-methyl-4-dimethylaminoazobenzene (Miller *et al.*, 1949) and in the livers of refractory rats after feeding 3′-methyl-4-dimethylaminoazobenzene (Griffin *et al.*, 1955 ; Ward and Spain, 1957 ; Spain and Clayton, 1958) suggests that the formation of bound dye in the liver may be a necessary, but not sufficient, requirement for the induction of liver tumours by aminoazo dyes (Miller and Miller, 1955).

Besides its presence in the liver, a relatively small amount of bound dye has also been detected in the blood plasma of rats fed 4-dimethylaminoazobenzene for two months (Miller and Miller, 1947). No further attention has, however, been given to the occurrence or possible importance of bound dye in blood or serum. We have, therefore, undertaken a study of this aspect of the problem. The present work reports the determination of bound dye in the serum and observations on the protein fractions to which it is attached.

### MATERIAL AND METHODS

*Reagents.*—3′-Methyl-4-dimethylaminoazobenzene (3′-MeDAB ; m.p. 120·4–121·2° C.) was prepared by coupling diazotized *m*-toluidine with dimethylaniline as described by Miller and Miller (1948).

The borate-phosphate buffer used in the electrophoresis experiments had an ionic strength of 0·05 and contained 2·34 g. $NaH_2PO_4 . 2H_2O$ and 4·29 g. $Na_2B_4O_7 . 10\ H_2O$ per litre. The pH was 8·45 at 22° C.

*Treatment of animals.*—Male albino rats (weight 180–220 g.) of the Wistar strain were provided by the National Nutrition Research Institute, Pretoria. The animals were housed in wire cages at 20°C. and fed *ad libitum* on the Institute's stock diet (protein, 20 per cent ; ash, 7·4 per cent ; main component : maize meal, 56 per cent) and tap water.

A single dose of 50 mg. of 3′-MeDAB in 2 ml. of olive oil was administered by stomach tube to rats which were fasted for four hours before dosing, and their blood was drawn 45 hours later. Preliminary tests had shown that the level of bound dye in the serum and the liver reached a maximum at about 40 hours after dosing.

For the collection of serum, the animals were anaesthetized with ether, the abdominal cavities exposed and the blood was obtained from the abdominal aorta. The blood was left to clot, centrifuged and the serum obtained.

*Column electrophoresis.*—Column electrophoresis of the serum was performed as described by Gedin and Porath (1957) in a vertical column (diameter 1·75 cm., length 150 cm.) filled with Munktell's cellulose powder. The column was cooled with water of 4° C. in the outer jacket. The serum was dialysed against two changes of buffer (1 litre). Eight ml. of dialysed serum (i.e. about 500 mg. of protein) was applied to the column. Using a voltage of 1500 V and a current of 25 mA the electrophoresis was carried out for 52 hours. After electrophoresis, the separated proteins were displaced from the column with buffer and the effluent was collected in fractions of 3·8 ml. The protein content of each fraction was determined by measuring the absorption at 280 m$\mu$. The optical densities were plotted against the numbers of the fractions and the resulting pattern was used as a guide for combining the fractions to obtain five serum protein fractions, namely, pre-albumin, albumin, $\alpha$-globulin, $\beta$-globulin and $\gamma$-globulin. These combined fractions were dialysed free from salts against distilled water, freeze-dried and then dried *in vacuo* over $P_2O_5$. The identity of these fractions was ascertained by paper electrophoresis on horizontal strips of Whatman 3 MM filter paper.

*Estimation of dye.*—For the determination of free, polar bound and non-polar bound dye in the serum and in the serum protein fractions, a modification of the methods of Miller and Miller (1947) and of Ward and Spain (1957) was used.

The free dye was extracted from salt-free, lyophilized protein samples (50–80 mg.) with 50 ml. of absolute ethyl alcohol for 15 minutes at 75° C. After centrifugation the residue was re-extracted twice with a boiling mixture of 30 ml. of absolute alcohol and 10 ml. of peroxide-free diethyl ether. Determination of free dye in each subsequent extract proved that this procedure gave quantitative extraction of the free dye. The extracts were combined, concentrated to 20 ml., chilled, 25 ml. of chilled 7 N HCl added, and the optical absorption measured after 15 minutes at 505 m$\mu$, using a 10 cm. absorption cell. The blank correction was obtained after reduction of the azo dye by addition of 0·45 ml. of a saturated solution of stannous chloride in concentrated hydrochloric acid.

After these extractions the proteins were dried *in vacuo* over $P_2O_5$. A weighed amount of the dried protein was hydrolysed in an aqueous KOH-alcohol mixture and the non-polar and polar bound dyes were determined according to the method of Miller and Miller (1947). Blank corrections were again obtained after reduction with stannous chloride.

The results of the measurements have been calculated as m$\mu$moles of azo dye per 100 mg. of protein, as described by Ward and Spain (1957).

Control experiments in which 0·3 mg. of 3′-MeDAB was added to 5 ml. of normal rat serum and allowed to stand overnight at 4° C. before extraction, showed that no *in vitro* formation of bound dye either in the polar or non-polar form occurred under the above conditions.

*Spectrum of bound dye.*—The absorption spectra of polar bound dye obtained from the liver (according to the method of Ward and Spain, 1957) as well as from the serum of the same rats were obtained as the difference between the spectra in ethyl-alcohol-7 *N* hydrochloric acid (4 : 5) before and after reduction with stannous chloride.

### RESULTS

Free electrophoresis of serum obtained 45 hours after administration of 50 mg. of 3′-MeDAB showed that the pattern of the serum proteins was completely normal.

The amounts of free and non-polar and polar bound dye in two sera, each obtained from three rats 45 hours after administration of 50 mg. of 3′-MeDAB, are shown in Table I. From this it is seen that in addition to the free dye, appreciable amounts of polar bound dye were present. The non-polar bound dye concentration, however, was small.

TABLE I.—*Azo Dye Concentrations in Sera of Rats after Administration of 50 mg. of 3′-MeDAB. Concentrations are Expressed as m$\mu$moles Dye per 100 mg. Protein*

| | Serum A | Serum B |
|---|---|---|
| Free dye | 81 | 95 |
| Non-polar bound dye | 2 | 2 |
| Polar bound dye | 20 | 36 |

The elution curves obtained after column electrophoresis of two sera, A and C, each from three rats treated with 50 mg. of 3′-MeDAB showed a significant pre-albumin component. According to Garbers and Joubert (1958) this effect is due to an increased electrophoretic mobility of $\alpha_1$-glycoprotein in the complexing borate buffer used. From analysis of the elution curves it was estimated that the pre-albumin fractions used for aminoazo dye determinations contained up to 50 per cent of albumin. The relative proportions of the protein fractions (Table II) were obtained from the areas of the corresponding portions of the elution curves.

The amounts of dye associated with the serum protein fractions are also given in Table II. The quantities of $\alpha$-globulin available for the dye determinations were small, and consequently the values calculated for the amount of dye in this protein fraction are less reliable.

TABLE II.—*Azo Dye in Protein Fractions of Rat Serum After Administration of* 50 *mg. of* 3′-*MeDAB*

| Serum | Serum protein fraction | Serum protein composition (per cent of total serum protein) | Concentration of dye in fraction (mμmoles/100 mg. protein) Free dye | Non-polar bound dye | Polar bound dye | mμmoles polar bound dye in protein fractions of 100 mg. total serum protein (calculated) |
|---|---|---|---|---|---|---|
| A | Pre-Albumin | 17 | 5 | 1 | 14 | 2·4 |
| | Albumin | 37 | 101 | 2 | 30 | 11·7 |
| | α-globulin | 7 | (4) | 0 | (3) | 0·2 |
| | β-globulin | 20 | 0 | 0 | 3 | 0·6 |
| | γ-globulin | 18 | 7 | 0 | 3 | 0·5 |
| C | Pre-Albumin | 16 | 0 | 1 | 11 | 1·8 |
| | Albumin | 33 | 12 | 1 | 22 | 7·3 |
| | α-globulin | 11 | 0 | 1 | (4) | 0·4 |
| | β-globulin | 20 | 0 | 0 | 2 | 0·4 |
| | γ-globulin | 20 | 0 | 0 | 3 | 0·6 |

The free dye in the serum is associated essentially with the albumin. However, only insignificant amounts of free dye could be detected in the pre-albumin fractions, although a large part of these fractions is albumin. It therefore appears that the free dye is adsorbed on the slower moving albumin.

Relatively large amounts of polar bound dye are associated with the albumin, and only very small amounts of polar dye seem to be bound to α-globulin, β-globulin and γ-globulin. The polar dye found in the pre-albumin fraction may be accounted for as albumin-bound dye. Calculation of the total amount of polar dye bound to each protein fraction per 100 mg. of total serum proteins shows that approximately 90 per cent of the polar dye in the serum is bound to albumin (Table II, last column).

In order to compare the protein-bound dyes found in the serum to those found in the liver, the absorption spectra of the dyes released in either case, by alkaline hydrolysis, have been measured in acid-ethyl alcohol solution. These spectra were of similar shape in the wavelength range 400 mμ to 600 mμ, but the maximum occurred at 520 to 522 mμ in the case of dye released from serum or serum albumin and at 525 mμ in the case of that from liver protein.

### DISCUSSION

It is known that most of the free (demethylated) aminoazo dyes in the blood are associated with the erythrocytes (Miller, Miller and Baumann, 1945). The demonstration of free dye in the serum of rats fed a single dose of 50 mg. 3′-MeDAB is, therefore, of interest because similar quantities of free 3′-methyl-4-aminoazobenzene have been found in the blood of rats fed 3′-MeDAB in the diet for 15–33 days (Miller *et al.*, 1958).

Of greater importance is the present demonstration of substantial amounts of polar bound dye in the serum. The dye released from serum proteins by alkaline hydrolysis is similar in solubility properties (e.g. in petroleum-ether and ethanol-ether mixtures) to that bound to liver proteins in the same rats. The absorption spectra were also generally similar, except that a small difference in wavelength of maximum absorption was observed. The significance of this difference is uncertain. Terayama *et al.* (1958) and Terayama, Kusama and Aoki (1958) fractionated protein-bound DAB derivations after protein hydrolysis into a fraction with $\lambda_{max}$ 514 m$\mu$ and another with $\lambda_{max}$ 522 m$\mu$ and they considered that the first fraction contained a primary amino group, and the second a secondary amino group. The question whether similar differences exist between the dyes bound to serum and liver proteins cannot be answered at this stage.

Unpublished work in this laboratory has shown that the amount of bound dye per 100 mg. of protein in the serum is approximately half that in the liver of the same rats at all times between 12 and 45 hours after feeding. Taking into account the relative weights of liver and serum proteins in the rat, the total quantity of bound dye in the serum is approximately one-quarter of that in the liver. This constitutes evidence that the appearance of bound dye in the serum parallels that in the liver. From calculations based on a half-life of $3\frac{1}{2}$ days for the liver protein-bound dye (Miller and Miller, 1952 ; Gelboin *et al.*, 1958) it also appears unlikely that the considerable amount of bound dye in the serum has been formed from the liver protein-dye complex. It should furthermore be noted that the bound dye in the serum is associated with the electrophoretically fast moving albumin fraction, while in the liver the bulk of the bound azo dye has been found in a slow moving fraction of the soluble proteins (Sorof *et al.*, 1951).

The demonstration that the bound dye in the serum is associated with the albumin fraction is of interest in view of the important role of the microsomes of liver cells in the process of albumin synthesis (Peters, 1957, 1959 ; Campbell, Greengard and Kernot, 1960) as well as in the process of incorporation of aminoazo dyes into proteins during their synthesis (Hultin, 1956, 1957 ; Gelboin *et al.*, 1958).

These considerations suggest that the binding of aminoazo dyes is not a highly specific process, involving a single protein, but rather that it tends to occur on a number of proteins (see also Terayama and Otsuka, 1960).

The relative importance of incorporation of various aminoazo dyes into liver and serum proteins and the mechanism of binding is now being studied in this laboratory.

SUMMARY

Significant quantities of free and bound dye have been observed in the serum of rats fed a single dose of 3′-methyl-4-dimethylaminoazobenzene. After column electrophoresis on cellulose, these dyes were shown to be associated with the albumin fraction. The results do not support the contention that binding of aminoazo dye to protein in the liver is highly specific.

The authors wish to thank Dr. H. M. Schwartz for her interest and valuable discussions, and Mr. J. J. Dreyer and his staff of the Physiology Department of the National Nutrition Research Institute for their kind co-operation in providing all the animals.

This project was sponsored by the National Cancer Association of South Africa.

### REFERENCES

Campbell, P. N., Greengard, O. and Kernot, B. A.—(1960) *Biochem. J.*, **74**, 107.
Garbers, C. F. and Joubert, F. J.—(1958) *Nature, Lond.*, **182**, 530.
Gedin, H. I. and Porath, J.—(1957) *Biochim. biophys. Acta*, **26**, 159.

GELBOIN, H. V., MILLER, J. A. AND MILLER, E. C.—(1958) *Cancer Res.*, **18**, 608.
GRIFFIN, A. C., RICHARDSON, H. L., ROBERTSON, C. H., O'NEAL, M. A. AND SPAIN, J. D.—(1955) *J. nat. Cancer Inst.*, **15**, 1623.
HULTIN, T.—(1956) *Exp. Cell Res.*, **10**, 71.—(1957) *Ibid.*, **13**, 47.
MILLER, E. C. AND MILLER, J. A.—(1947) *Cancer Res.*, **7**, 468.—(1952) *Ibid.*, **12**, 547.—(1955) *J. nat. Cancer Inst.*, **15**, 1571.
*Idem*, MILLER, J. A., BROWN, R. R. AND MACDONALD, J. C.—(1958) *Cancer Res.*, **18**, 469.
*Idem*, MILLER, J. A., SAPP, R. W. AND WEBER, G. M.—(1949) *Ibid.*, **9**, 336.
MILLER, J. A. AND MILLER, E. C.—(1948) *J. expt. Med.*, **87**, 139.—(1953) *Advanc. Cancer Res.*, **1**, 339.
IIDEM AND BAUMANN, J. A.—(1945) *Cancer Res.*, **5**, 162.
PETERS, T., JR.—(1957) *J. biol. Chem.*, **229**, 659.—(1959) *J. Histochem. Cytochem.*, **7**, 224.
SOROF, S., COHEN, P. P., MILLER, E. C. AND MILLER, J. A.—(1951) *Cancer Res.*, **11**, 383.
SPAIN, J. D. AND CLAYTON, C. C.—(1958) *Ibid.*, **18**, 155.
TERAYAMA, H., ISHIDATE, M. AND HANAKI, A.—(1959) *Nature, Lond.*, **184**, 1460.
*Idem*, KUSAMA, K., TERUYA, K., KURODA, S. AND NAKAYAMA, T.—(1958) *Gann*, **49**, 85.
*Idem*, KUSAMA, K. AND AOKI, T.—(1958) *Ibid.*, **49**, 97.
*Idem* AND OTSUKA, M.—(1960) *Ibid.*, **51**, 113.
WARD, D. N. AND SPAIN, J. D.—(1957) *Cancer Res.*, **17**, 623.


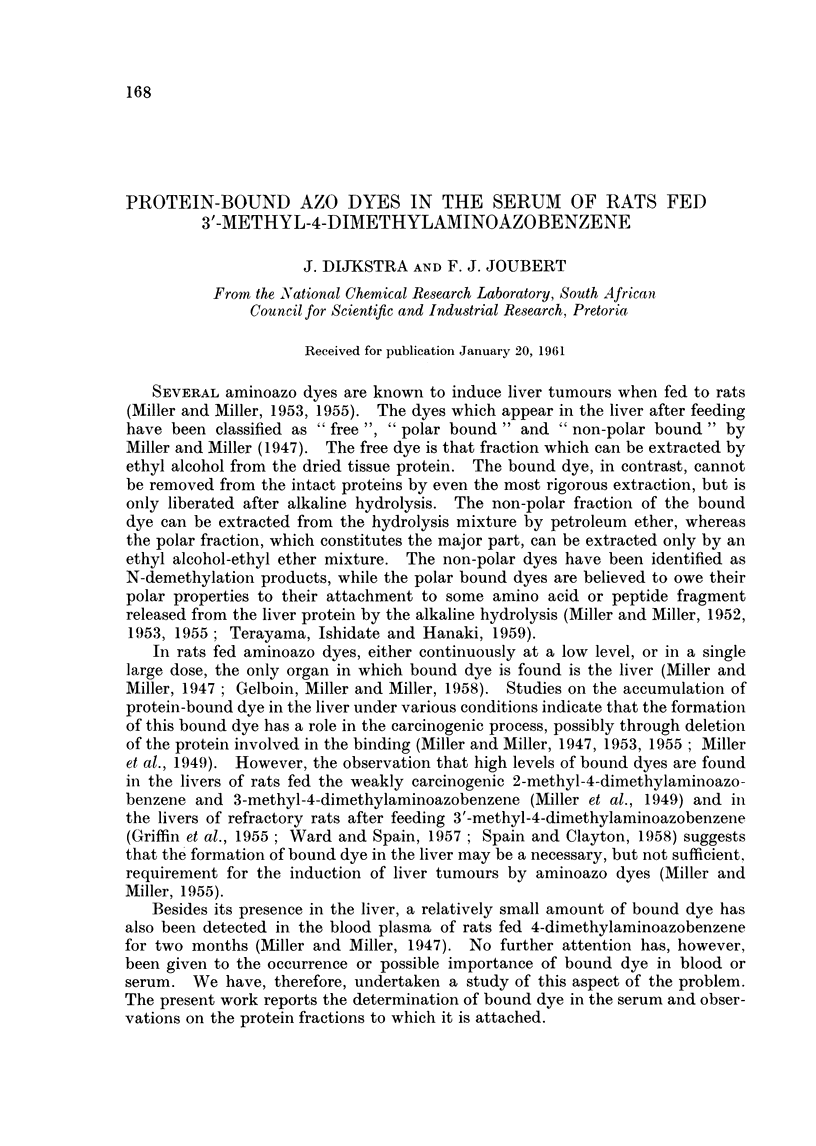

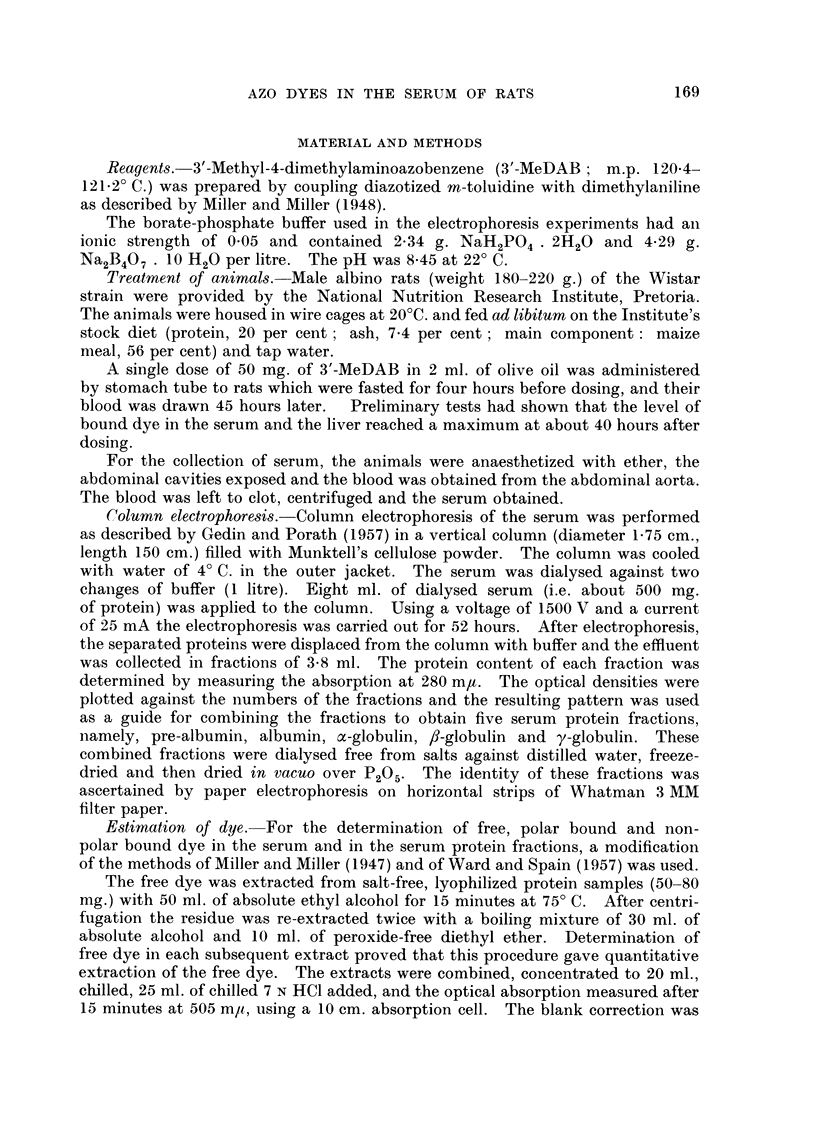

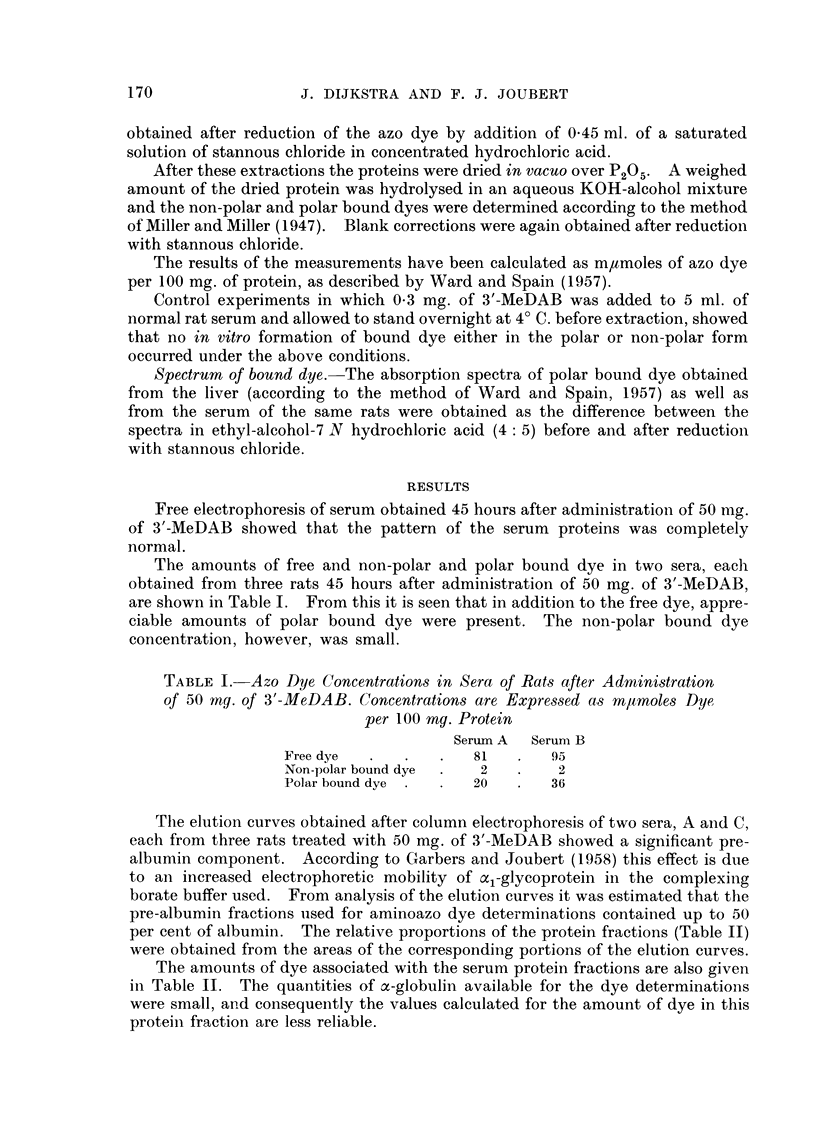

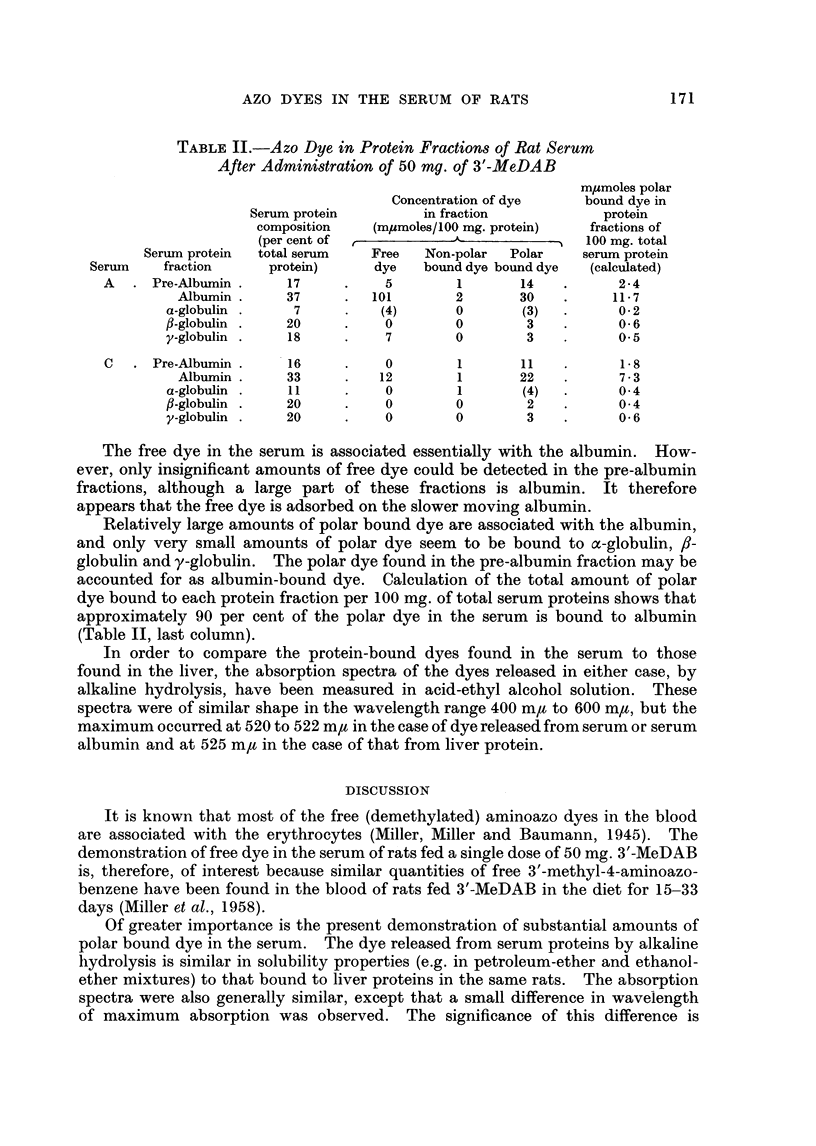

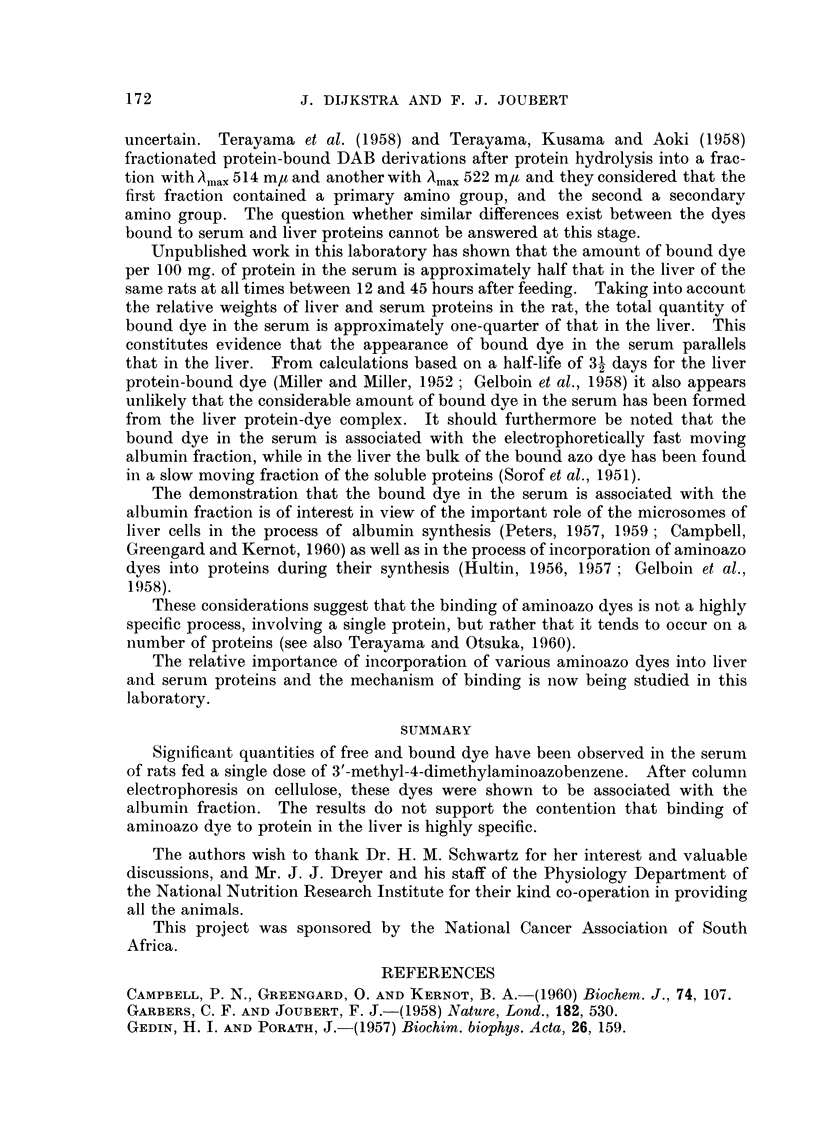

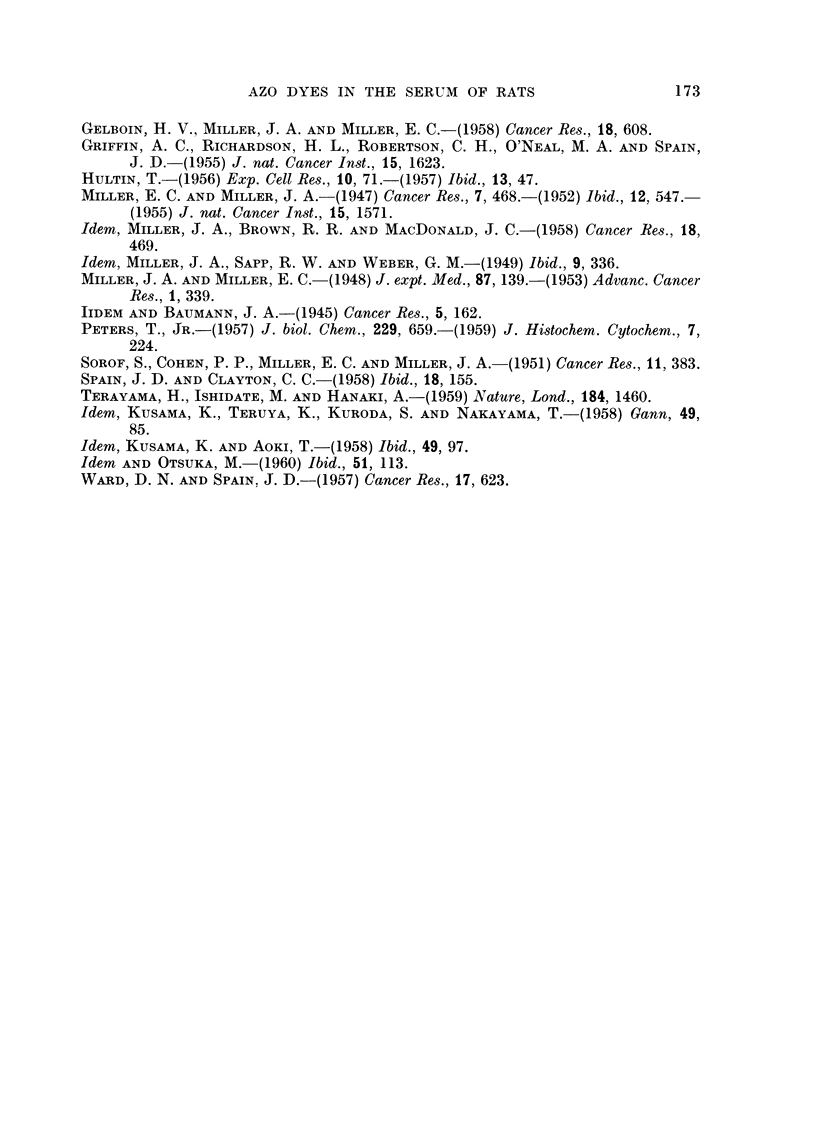


## References

[OCR_00303] GARBERS C. F., JOUBERT F. J. (1958). Effect of the borate ion in buffers on the electrophoresis of rat serum.. Nature.

[OCR_00305] GEDIN H. I., PORATH J. (1957). Studies of zone electrophoresis in vertical columns. II. Zone electrophoresis of serum proteins.. Biochim Biophys Acta.

[OCR_00309] GELBOIN H. V., MILLER J. A., MILLER E. C. (1958). Studies on hepatic protein-bound dye formation in rats given single large doses of 3'methyl-4-dimethylaminoazobenzene.. Cancer Res.

[OCR_00311] GRIFFIN A. C., RICHARDSON H. L., ROBERTSON C. H., O'NEAL M. A., SPAIN J. D. (1955). The role of hormones in liver carcinogenesis.. J Natl Cancer Inst.

[OCR_00317] MILLER E. C., MILLER J. A. (1955). Biochemical investigations on hepatic carcinogenesis.. J Natl Cancer Inst.

[OCR_00333] PETERS T. (1957). A serum albumin precursor in cytoplasmic particles.. J Biol Chem.

[OCR_00337] SOROF S., COHEN P. P., MILLER E. C., MILLER J. A. (1951). Electrophoretic studies on the soluble proteins from livers of rats fed aminoazo dyes.. Cancer Res.

[OCR_00338] SPAIN J. D., CLAYTON C. C. (1958). Inhibition of azo dye carcinogenesis by thorotrast and iron oxide.. Cancer Res.

[OCR_00340] TERAYAMA H., ISHIDATE M., HANAKI A. (1959). Incorporation of Nmethyl carbon of monomethyl-aminoazodyes. into polardyes.. Nature.

[OCR_00349] WARD D. N., SPAIN J. D. (1957). Protein-bound azo dye in the liver of hypophysectomized rats.. Cancer Res.

